# Struct2SL: Synthetic lethality prediction based on AlphaFold2 structure information and Multilayer Perceptron

**DOI:** 10.1016/j.csbj.2025.04.012

**Published:** 2025-04-12

**Authors:** Yurui Huang, Ruzhe Yuan, Yaxuan Li, Zheming Xing, Junyi Li

**Affiliations:** School of Computer Science and Technology, Harbin Institute of Technology (Shenzhen), Shenzhen, Guang Dong 518055, China

**Keywords:** Synthetic lethality prediction, AlphaFold2 protein structure, Multilayer perceptron, Network link prediction

## Abstract

In cancer therapeutics, the elucidation of synthetic lethality principles introduces transformative concepts for devising novel treatment paradigms. Computational methods to predict synthetic lethal (SL) gene pairs have potential to markedly enhance the precision and efficacy of cancer interventions. Despite the array of predictive methodologies proposed in extant research, many overlook pivotal attributes such as protein sequences, three-dimensional configurations, and protein-protein interaction (PPI) networks. This investigation introduces Struct2SL, a predictive framework for SL gene pairs that integrates protein sequences, PPI networks, and three-dimensional protein structures. By initiating at the protein feature stratum, Struct2SL offers a novel vantage point to refine the feature representation of gene interactions, thereby enabling more accurate predictions of prospective SL pairs. Struct2SL encompasses four distinct phases: Initially, protein three-dimensional structures, sequence characteristics, and interaction network attributes are extracted utilizing approaches such as Alphafold2 for predicting protein tertiary structures. Subsequently, the preliminary embedding of genes is derived by consolidating information via the protein-gene mapping relationships. Thereafter, an SL graph is constructed to attain the ultimate gene embedding. Ultimately, a multilayer perceptron is employed for the prediction of SL interactions. The outcomes indicate that Struct2SL outperforms four SOTA methods, as gauged by the evaluation metrics. This implies that Struct2SL is more efficacious in predicting SL gene pairs. This study furnishes a new and efficient computational approach for the prediction of SL gene pairs in cancer therapy, anticipated to catalyze advancements in the field of oncological treatment. We also developed a webserver (Synthetic Lethality Query Server, http://struct2sl.bioinformatics-lilab.cn) to present cancer synthetic lethal genetic interactions, which is designed to provide researchers with an accessible tool for predicting synthetic lethality gene pairs.

## Graphical Abstract

**Figure:**
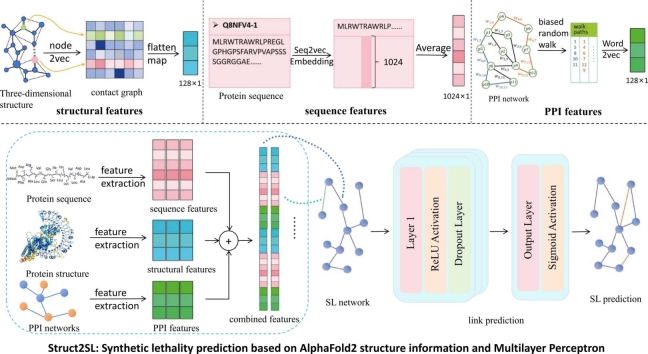

## 1 Introduction

In cancer treatment, molecular biology has illuminated the potential for targeted therapies, where precision strikes against tumor cells are mediated through the engagement of specific molecular pathways [1]. The concept of synthetic lethality (SL) has introduced a paradigm shift in cancer treatment strategies. Synthetic lethality[2] is characterized by a scenario wherein the mutation of either of two genes in isolation does not precipitate cellular demise, yet the concurrent mutation of both genes is lethal to the cell. Capitalizing on this biological phenomenon, pharmacological interventions can be engineered to selectively target and incapacitate tumor cells that harbor specific genetic aberrations, whilst sparing normal cells from harm. The swift advancement of high-throughput experimental methodologies has led to the identification of an expanding repertoire of SL gene pairs, thereby presenting a burgeoning array of potential therapeutic targets for cancer therapeutics. The predominant laboratory screening modalities encompass RNA interference (RNAi) technology[3], which elucidates gene function through the targeted silencing of gene expression, and CRISPR gene-editing technology[4], which probes the functional nuances of genes by effectuating precise genomic alterations. However, the inherent limitations of these laboratory-based screening techniques, such as prohibitive costs, protracted experimental timelines, and the potential for eliciting undesired side effects, have spurred the scientific community to pivot towards the development of computational approaches for the prediction of SL gene pairs. This computational initiative is poised to efficaciously complement and enhance the capabilities of traditional laboratory testing protocols.

To surmount the constraints imposed by experimental screening methodologies, researchers have initiated the computational prediction of synthetic lethal (SL) gene pairs. Computational predictive techniques [5] confer several advantages, such as reduced costs and accelerated processing times. These methods can substantially augment experimental screening procedures. Based on the types of data and algorithms utilized, computational prediction methods can be categorized into two principal classes: those predicated on domain knowledge and those founded on machine learning.

Computational methods that are anchored in domain expertise harness extant biological information and datasets to discern SL gene pairs. These approaches depend on a profound comprehension of biology to yield contextually pertinent predictions, employing data mining techniques and statistical analyses to accurately predict gene interactions culminating in synthetic lethality. They generally necessitate researchers to possess a certain degree of domain-specific knowledge to appropriately interpret and apply such insights. For instance, the DAISY method[6] utilizes statistical analyses grounded in gene expression data to pinpoint SL gene pairs. The MiSL method[7] leverages cancer genomic information to identify SL gene partners that are specific to particular cancer types, with a focus on mutations within these datasets. The strength of these methods lies in their utilization of existing biological data and knowledge; however, they are bounded by the scope of known data and exhibit limited predictive efficacy for undiscovered SL gene pairs.

Computational methods which are based on machine learning extrapolate gene features from voluminous datasets and construct predictive models to forecast SL gene pairs. For example, the SL2MF method[8] derives gene features from the gene differential expression similarity matrix via matrix decomposition technology to predict potential SL interactions. The DiscoverSL method [9] amalgamates multiple data sources, such as mutation data and gene expression data, by constructing a multi-parameter random forest classifier to predict synthetic lethal gene pairs in specific cancers. Moreover, the PARIS algorithm[10] forecasts synthetic lethal interactions in cancer cells by scrutinizing CRISPR-Cas9 gene editing screening data and integrating genomic and transcriptomic information. The advantage of these methods is their capacity to process large-scale datasets; however, they necessitate manual feature engineering and are vulnerable to noisy data. Machine learning methods are more data-centric, concentrating on pattern recognition and feature extraction from extensive datasets without an obligatory reliance on explicit biological knowledge.

Deep learning methods facilitate the construction of complex network architectures, which are capable of automatically extracting salient features from data, effectively capturing the nuanced interactions among genes. The DDGCN model [11] was pioneering in applying GCN to the realm of SL prediction. It integrates a graph convolutional strategy with a dual dropout mechanism to address the challenge of sparse data and to reduce the risk of overfitting. The PiLSL method [12] constructs a closed subgraph for each gene pair and incorporates an attention-based embedding propagation layer to evaluate the significance of edges within the subgraph. The TARSL method [13] captures the correlation between molecular features and network connections through feature-level attention, discerns the importance of different neighboring nodes through node-level attention, and focuses on significant networks through network-level attention. The KG4SL model [14], based on the knowledge graph (KG), learns gene representations by sampling subgraphs around individual genes within the KG. The KR4SL method [15] builds upon the KG4SL concept by constructing a relational directed graph to capture the structural semantics embedded within the knowledge graph. The SLGNN model [16] focuses on gene preferences across diverse relationships in the knowledge graph and designs a graph neural network (GNN) architecture to efficiently learn gene representations.

These methods have demonstrated promising results in SL prediction, yet they predominantly rely on the representation of genetic data features for making predictions. DeepMind's AlphaFold [17] represents a transformative advancement in the prediction of protein structures. Drawing on this, we have proposed an AlphaFold-based approach, Struct2SL, which comprehensively considers the characteristic representation performance of protein sequences, protein-protein interaction (PPI) networks, and protein 3D structures on gene interactions, thereby enhancing the prediction of potential SL pairs. Our model encompasses four stages: Initially, we employ methods such as AlphaFold2 (version 2.3.1) to predict the 3D structure of proteins, thereby extracting protein 3D structure, sequence features, and interaction network features. Subsequently, we leverage the correlation between proteins and genes to compile the proteins' explicit data, resulting in a preliminary gene representation. Next, we utilize established SL gene pairs to form SL graphs, which yield the final gene embedding. Finally, we apply a multilayer perceptron to train the model for predicting SL interactions. In comparison, the Struct2SL outperforms five other state-of-the-art methods.

## 2 Datasets and materials

In the context of synthetic lethality prediction, the scope of model applicability is a critical factor. We opted to train our model in a pan-cancer setting rather than a cell-specific one for several reasons. First, a pan-cancer approach enables the model to capture generalizable patterns and relationships across multiple cancer types, identifying synthetic lethality interactions that are relevant across different cancers rather than being confined to specific cell lines. Second, pan-cancer datasets offer a larger and more diverse pool of data, which enhances the robustness and generalizability of the model. In contrast, cell-specific datasets are typically smaller and may lack the diversity needed for comprehensive training. Finally, from a clinical standpoint, identifying SL interactions that apply across multiple cancers can have a broader impact, potentially leading to therapeutic strategies that target multiple cancer types.

The datasets utilized in this study are predominantly sourced from four public databases: Syn-LethDB2.0 [18], UniProt [19], STRING [20], and AlphaFold DB [21]. These databases collectively offer a wealth of data essential for the prediction of synthetic lethal gene pairs.

Syn-LethDB is a comprehensive repository of synthetic lethality data, providing gene pairs associated with synthetic lethality across various organisms, including humans. For this study, human synthetic lethality data were selected, excluding pairs derived solely from computational predictions. This dataset encompasses 9856 genes and 26,614 synthetic lethal gene pairs, forming the cornerstone for model training and validation.

UniProt serves as a premier resource for protein information, offering detailed insights including protein sequences and functional annotations. A total of 20,504 experimentally confirmed human protein sequences were retrieved from UniProt for this study. These sequence details are pivotal for elucidating the structural and functional aspects of proteins.

STRING is a widely recognized database for protein interactions, furnishing information on functional associations, interactions, and metabolic pathways among proteins. Human protein interaction network data, comprising 17,740 proteins and 1477,610 interaction relationships, were obtained from STRING. These interaction data are crucial for deciphering the intricate relationships between proteins.

AlphaFold DB is an open-source, deep learning-powered model designed to predict the spatial configuration of proteins. It is capable of inferring a protein's 3D structure from its amino acid sequence with a precision often comparable to that achieved through experimental methods. For this study, the three-dimensional structural data of 23,391 human proteins were sourced from the AlphaFold protein structure database.

## 3 Model and implementation

### 3.1 Overview of Struct2SL

**Fig. 1:**
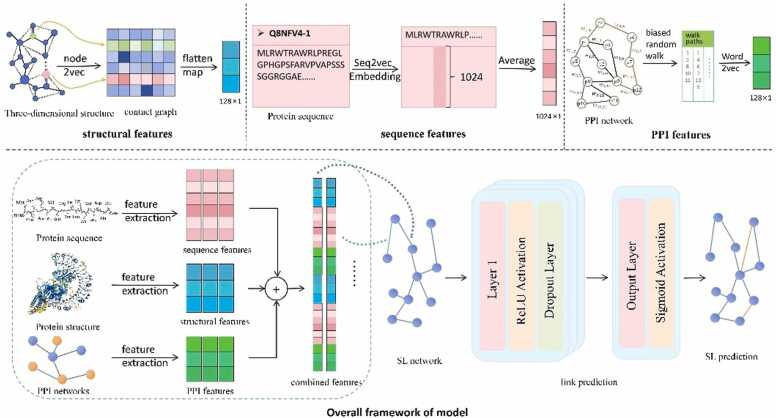
Overall framework of the model Struct2SL.

In the present study, we have developed a framework leveraging Multilayer Perceptron to automatically distill gene feature embeddings from protein sequence, structural, and protein-protein interaction (PPI) network data. This framework encapsulates gene interactions and functional correlations by amalgamating diverse bioinformatics data sources. The architecture of the model is depicted in Fig. 1. Initially, advanced deep learning methodologies, namely seq2vec and node2vec, are employed to generate feature embeddings from protein sequences and PPI networks. Subsequently, the gene feature representations are augmented through the computation of protein three-dimensional structural attributes. Furthermore, to enhance the diversity and size of the dataset, a data augmentation strategy using negative random sampling techniques is implemented to generate reliable negative data. Ultimately, harnessing the link prediction capabilities of the MLP model, we discern prospective synthetic lethal gene pairs.

### 3.2 Feature extraction

#### 3.2.1 Protein sequence characteristics

In the protein sequence feature processing phase, the seq2vec method, which is grounded in deep learning, is utilized to autonomously extract semantic information from protein sequences and to generate corresponding feature embeddings. This approach circumvents the intricacies associated with manual feature design by capitalizing on pre-trained deep learning models to discern complex patterns inherent in sequences.

ELMo (Embeddings from Language Models) is a pre-trained deep bidirectional language model designed to capture the semantics of sequential data. The model is trained on large corpora to learn contextualized word embeddings that can dynamically adapt to different contexts. The pre-trained model parameters used in this study are sourced from AllenNLP, which is an open-source natural language processing library that provides a range of pre-trained models and tools for researchers and developers to build and experiment with NLP models.

The seq2vec transformation begins with constructing a sequence dictionary from the protein sequence file. A function for processing embeddings is then defined. This function processes the embedding representation produced by the ELMo model and computes the mean of all time steps to yield a singular embedding vector for each protein. Specifically, the embedding vector ${e_{i}}^{\left(0\right)}$ for the *i*-th protein is calculated as:

(3-1) $$\begin{array}{c} {e_{i}}^{\left(0\right)}\;=\;\frac{1}{T}\sum_{t=1}^{T}h_{t} \end{array}$$

Herein, $h_{t}$ represents the hidden state at time step *t*, and *T* denotes the sequence length. This operation effectively averages the contextualized embeddings across the entire sequence to obtain a fixed-size representation for each protein.

Subsequently, the embedding representation layer is iteratively updated to acquire higher-order features. This process involves aggregating information from neighboring entities to enrich the representation of each entity.

Specifically, the embedding representation of entity *i* at layer *l+ 1* is updated as:

(3-2) $$\begin{array}{c} e_{i}^{\left(l+1\right)}=\mathrm{aggregate}(e_{i}^{\left(l\right)},{\{e}_{j}^{\left(l\right)}|j\in N(i)\}) \end{array}$$

In this context, $e_{i}^{\left(l\right)}$ signifies the embedding representation of entity *i* at layer *l*, $N\left(i\right)$ represents the set of neighboring entities of entity *i*, and *aggregate* is a function that amalgamates information from both the entity itself and its adjacent entities.

The aggregation method used in this study is average aggregation, which averages the embedding vectors of all residues of each protein to obtain a fixed-size protein embedding vector.

Ultimately, the embedding representations from all layers are aggregated to derive the final protein embedding. The final embedding vector $e_{i}^{\mathrm{final}}$ for the *i*-th protein is obtained by summing the embeddings from all layers:

(3-3) $$\begin{array}{c} e_{i}^{\mathrm{final}}=\sum_{l=0}^{L}e_{i}^{\left(l\right)} \end{array}$$

Here, *L* indicates the total number of layers in the network. This final embedding vector encapsulates the hierarchical information from all layers, providing a comprehensive representation of the protein sequence that can be utilized for downstream analysis.

#### 3.2.2 PPI characteristics

In the phase of processing protein-protein interaction (PPI) network data, a deep learning-based approach, node2vec[22], was employed to automatically extract semantic information from nodes (proteins) within the network and to generate feature embeddings. This method adeptly captures both the network's topological structure and the neighborhood information of the nodes.

The node2vec methodology explores the network through simulated random walks to acquire the embedding representation of the nodes. Initially, a starting node is selected from the network, and a random walk is conducted according to a predefined transition probability. This probability is calculated by considering the neighborhood of the current node along with parameters *p* and *q*. Specifically, for each neighbor of the current node, the probability of it being selected as the next node is computed, which is proportional to the neighbor's weight and is modulated by parameters *p* and *q*. Mathematically, the transition probability can be expressed as:

(3-4) $$\begin{array}{c} P\left(v_{t+1}=u|,v_{t}=v\right)=\left\{ \begin{array}{cc} \frac{1}{p}\cdot \frac{w_{\mathrm{uv}}}{\sum_{x\in N\left(v\right)}w_{\mathrm{vx}}} & \mathrm{if}\;u\in N\left(v\right)\;\;\mathrm{and}\;\;u\neq v_{t-1}\\ \frac{1}{q}\cdot \frac{w_{\mathrm{uv}}}{\sum_{x\in N(v)\backslash \{v_{t-1}\}}w_{\mathrm{vx}}} & \mathrm{if}\;u\in N\left(v\right)\;\;\mathrm{and}\;\;u\neq v_{t-1}\\ & \mathrm{and}\;\exists w\in N\left(u\right)\\ & \mathrm{such}\;\mathrm{that}\;\;w\neq v\\ \frac{w_{\mathrm{uv}}}{\sum_{x\in N(v)\backslash \{v_{t-1}\}}w_{\mathrm{vx}}} & \mathrm{otherwise} \end{array}\right. \end{array}$$

where $w_{\mathrm{uv}}$ represents the edge weight between nodes *u* and *v*, and $N\left(v\right)$ is the set of neighbors of node *v*.

Subsequently, multiple random walks are initiated from each node to generate a series of walk paths. Each walk path represents a sequence of nodes that encapsulate the neighborhood information of the node.

These walk paths are then utilized as inputs to the word2vec model to derive the embedding representation for each node. word2vec, a pre-trained model for generating word embeddings, acquires vector representations of words by optimizing the Skip-gram objective function. Here, the sequence of nodes in each walk path is treated similarly to a sequence of words in a sentence, and the embedding vector for each node is trained using this model.

Ultimately, the final node embedding is obtained by averaging the embedding representations for each node across all walk paths. These vectors encapsulate both the structural and semantic characteristics of the node within the network, effectively extracting salient features from the original PPI network data.

#### 3.2.3 Protein structural characteristics

In the phase dedicated to processing protein three-dimensional structural features, a sequence of computational and transformational procedures is employed to distill informative feature embeddings from the native three-dimensional structural data of proteins[23]. This encompasses the extraction of structural information, computation of interatomic distances, one-hot encoding of sequences, and the assembly of a contact matrix predicated on a distance threshold.

Initially, the coordinates of the Cα atoms within the protein structure are extracted. For each residue within the structure, this is represented as: $r_{i}=(x_{i},\;y_{i},\;z_{i})$, where $r_{i}$ symbolizes the coordinate vector of the Cα atom of the *i*-th residue. Utilizing the coordinates of the Cα atoms, the distances between all residue pairs are computed, culminating in the construction of the distance matrix *D*:

(3-5) $$\begin{array}{c} D_{\mathrm{ij}}=\sqrt{{\left(x_{i}-x_{j}\right)}^{2}{+\left(y_{i}-y_{j}\right)}^{2}+{\left(z_{i}-z_{j}\right)}^{2}} \end{array}$$

Here, $D_{\mathrm{ij}}$ signifies the Euclidean distance between the *i*-th and *j*-th residues.

Subsequently, the protein sequence is transcribed into a one-hot encoded format to preserve the compositional nuances of the sequence. For a sequence comprising *V* unique amino acids, the one-hot encoded vector for the *i*-th amino acid is delineated as:

(3-6) $$\begin{array}{c} S_{\mathrm{ij}}=\left\{ \begin{array}{cc} 1 & if\;\;\mathrm{the}\;\;i\mathrm{th}\;\;a\mathrm{mino}\;\;\mathrm{acid}\;\;\mathrm{is}\;\;\mathrm{the}\;\;\mathrm{jth}\;\;\mathrm{type}\\ 0 & \mathrm{otherwise} \end{array}\right. \end{array}$$

Thereafter, in accordance with the predefined threshold *cmap_thresh*, the distance matrix is transformed into an adjacency matrix *A* to depict the contact relationships within the protein structure:

(3-7) $$\begin{array}{c} A_{\mathrm{ij}}=\left\{ \begin{array}{cc} 1 & \mathrm{if}\;\;D_{\mathrm{ij}}<\mathrm{cmap}\_\mathrm{thresh}\\ 0 & \mathrm{otherwise} \end{array}\right. \end{array}$$

The adjacency matrix *A* and the one-hot encoding vector of the sequence are then amalgamated to formulate a graphical representation of the protein structure. This graphical representation is subsequently processed using the node2vec algorithm to generate feature embeddings that capture the structural context of each node within the graph. The node2vec algorithm performs biased random walks on the graph to explore the neighborhood of each node, generating sequences of nodes. The specific implementation details can be found in Section 3.2.2. This process results in a set of feature vectors that effectively encode the structural context of each node within the graph. The feature embeddings are then sorted and organized into a structured matrix format, yielding the final structural feature matrices that are used for further analysis.

For each protein, its structural features, sequence features, and PPI network node features are extracted. These three features are merged and subjected to normalization to obtain the functional features of each protein. Given that each protein is encoded by a primary gene, a one-to-one correspondence method is used to map gene features with protein features, thereby constructing the feature vector that describes the gene.

### 3.3 Data Augmentation through Random Negative Sample Generation

#### 3.3.1 Rationale for Random Negative Sample Generation

In the context of predicting synthetic lethal gene pairs, the inherent imbalance between positive and negative samples poses a significant challenge. Specifically, the dataset comprises 23749 positive SL pairs, whereas the number of negative samples is a mere 2745. In a comprehensive genomic landscape, the vast majority of gene pairs do not exhibit synthetic lethality. Consequently, the limited number of known negative samples is insufficient to provide a representative view of the non-SL gene pairs. To address this imbalance and to ensure that the machine learning models are trained on a more representative dataset, random negative sample generation is employed.

The principle of random negative sample generation is grounded in the assumption that the feature space of non-SL gene pairs can be adequately explored by randomly sampling pairs of genes that are not known to be SL pairs. This approach is particularly well-suited to the problem at hand, given the rarity of SL pairs and the vast number of possible gene pairs. By randomly selecting pairs of genes that are not in the set of known SL pairs, we can generate a larger and more diverse set of negative samples. This not only helps to balance the dataset but also ensures that the negative samples are representative of the broader population of non-SL gene pairs.

#### 3.3.2 Implementation of Random Negative Sample Generation

Initially, the set of valid genes is identified. These are genes that possess complete feature representations across all relevant feature types. This set of valid genes is denoted as *V*. Let *P* denote the set of positive samples (SL pairs) and *N* denote the set of known negative samples (non-SL pairs). Positive samples are processed first, followed by the addition of known negative samples. If the number of negative samples is insufficient to match the number of positive samples, new negative samples are generated through random selection, ensuring they do not overlap with existing pairs.

Random pairs of genes are selected from the set of valid genes *V*. Each pair $\left(g_{1},g_{2}\right)$ is then checked to ensure that it does not belong to the set of known SL pairs *P* or known non-SL pairs *N*. This step ensures that the generated negative samples are unique and representative of the non-SL gene pairs. It can be represented as:

(3-8) $$\begin{array}{c} \left(g_{1},g_{2}\right)\notin P\cup N \end{array}$$

Once a valid negative pair is identified, the feature vectors of the two genes are concatenated to form a combined feature vector, consistent with the process used for positive samples. This process continues iteratively until the number of negative samples reaches the desired balance with the positive samples. Following the augmentation process, the dataset undergoes standardization.

### 3.4 Multilayer Perceptron for Link Prediction

Given the fixed feature dimensions and the absence of spatial or temporal correlations in our data, we opted for a Multilayer Perceptron (MLP)-based model to predict potential links between synthetic lethal gene pairs. Synthetic lethality (SL) interactions are characterized by complex, non-linear relationships that stem from the intrinsic properties of the genes and their interactions within the cellular context. The MLP model is well-suited for this task, as it can effectively model these non-linear interactions and handle high-dimensional data. The core of this framework is to automatically extract complex relationship features between genes, thereby effectively capturing their interactions and functional associations.

The MLP model is constructed with multiple hidden layers, each equipped with a linear transformation followed by a non-linear activation function. In this study, the MLP model consists of three hidden layers with 256, 128, and 64 neurons, respectively. This configuration is chosen to progressively reduce the feature dimensions while capturing complex relationships between the input features. The architecture of the MLP can be formally described as follows:

Let $x_{i}^{\left(0\right)}$ be the initial feature vector of the *i*-th gene pair, where $W^{\left(k\right)}$ and $b^{\left(k\right)}$ are the weight matrix and bias vector of the *k*-th layer, respectively. The input to the first hidden layer is $h_{i}^{\left(0\right)}=x_{i}^{\left(0\right)}\;$. The output of the *k*-th hidden layer, $h_{i}^{\left(k\right)}$ is computed as:

(3-9) $$\begin{array}{c} \;h_{i}^{\left(k\right)}=\sigma_{h}\left(W^{\left(k\right)}h_{i}^{\left(k-1\right)}+b^{\left(k\right)}\right) \end{array}$$

where $\sigma_{h}$ is the non-linear activation function used in the hidden layers, specifically the ReLU function. For the output layer, a sigmoid activation function is used to predict the probability of a link:

(3-10) $$\begin{array}{c} \hat{y_{\mathrm{uv}}}=\sigma_{o}\left(W^{\left(L\right)}h_{i}^{\left(L-1\right)}+b^{\left(L\right)}\right) \end{array}$$

where $\sigma_{o}$ is the sigmoid activation function, and *L* is the total number of layers.

The link prediction model is trained using the binary cross-entropy loss function, which is appropriate for binary classification tasks:

(3-11) $$\begin{array}{c} L=-\frac{1}{N}\sum_{i=1}^{N}\left[y_{i}\log\left(\hat{y_{i}}\right)+\left(1-y_{i}\right)\log\left(1-\hat{y_{i}}\right)\right] \end{array}$$

where $y_{i}$ is the true label and $\hat{y_{i}}$ is the predicted probability. The model parameters are optimized using the Adam optimizer with a learning rate schedule that reduces the learning rate when the validation loss plateaus.

To mitigate the risk of overfitting, dropout regularization is implemented subsequent to each hidden layer. Specifically, a dropout rate of 50 % is applied, meaning that during each training iteration, 50 % of the neurons in each hidden layer are randomly dropped out. Dropout operates by randomly nullifying a proportion of the layer's output features during the training phase, a mechanism that aids in diminishing the co-adaptation of neurons. Additionally, L2 regularization is incorporated into the loss function to penalize large weights:

(3-12) $$\begin{array}{c} L_{\mathrm{reg}}=L+\lambda \sum_{k=1}^{L}{∥W^{\left(k\right)}∥}^{2} \end{array}$$

Where λ is the regularization strength. This regularization term helps in keeping the model weights small, thus reducing the model's complexity and improving its generalization ability.

Through the above method, we can automatically extract the complex relationship features between genes from the graph structure data and complete the link prediction task to identify synthetic lethal gene pairs. This approach not only boosts the efficiency of extracting features but also strengthens the model's capacity to forecast gene interactions.

## 4 Experiment Results

### 4.1 Baselines

To assess the effectiveness of Struct2SL, we conducted comparisons with several recently reported advanced methods for predicting SL interactions. The first method relies on graph convolutional networks, while the subsequent trio utilizes knowledge graph convolutional networks.

● DDGCN (Cai et al., 2020) predicts potential SL interactions using a GCN framework that incorporates a double dropout mechanism.
● KG4SL (Wang et al., 2021) pioneers the integration of knowledge graphs to enhance predictive models for synthetic lethal interactions.
● TARSL (Li et al., 2023) adopts a triple attention mechanism to integrate multimodal features in heterogeneous networks.
● SLGNN (Zhu et al., 2023) employs a knowledge graph neural network that accounts for gene preferences across various synthetic lethality-associated factors.

### 4.2 Selection of Positive and Negative Sample Ratio

The decision to utilize a 1:1 ratio of positive to negative samples in the model was driven by the goal of optimizing predictive accuracy. This section succinctly outlines the experimental rationale supporting this choice.

The experiments involved systematically varying the ratio of positive to negative samples and evaluating the model's performance using AUC, AUPR, and F1-score as key metrics. The results consistently demonstrated that the 1:1 ratio achieved the highest values for AUC, AUPR, and F1-score, which are crucial for assessing the model's ability to distinguish between SL and non-SL interactions. The results of the experiments are summarized in Table 2.

**Table 1:** Type and amount of data.

Types of data	Number of entities
Synthetic lethal gene pairs	23749
Nonsynthetic lethal gene pairs	2745
Protein sequence	20504
Protein-Protein Interaction Networks (nodes)	17740
Protein-Protein Interaction Networks (edges)	1477610
Protein spatial structure	23391

**Table 2:** Performance metrics for different positive to negative sample ratios (Struct2SL-m:n means the ratio of positive and negative samples in the model is m:n).

Methods	AUC	AUPR	F1
Struct2SL-1:1	**0.9812**±**0.0012**	**0.9798**±**0.0010**	**0.9399**±**0.0017**
Struct2SL-1:3	0.9799±0.0015	0.9604±0.0023	0.9106±0.0031
Struct2SL-1:5	0.9785±0.0009	0.9465±0.0036	0.8970±0.0039
Struct2SL-1:7	0.8705±0.1401	0.7267±0.2775	0.6018±0.3593

Taking into account the fact that the number of nonSL interactions is not less than that of SL interactions, the model opted for a 1:1 ratio. This choice is justified as it maximizes AUC, AUPR, and F1 scores, thereby yielding a model that more accurately, reliably, and efficiently predicts SL interactions. This balanced approach lays a robust foundation for further research and practical applications, ensuring that the model is well-equipped to perform the further research and application.

### 4.3 Model Evaluation

Struct2SL utilizes Python 3.9.19 and PyTorch 2.3.1^2^ as its principal development environments. The dataset is divided into training, validation and test sets, using a 5-fold cross-validation method with a random assignment ratio of 6.4:1.6:2 for each fold. Moreover, random negative sampling is employed to expand the non-synthetic lethal (non-SL) dataset, thereby producing an equal number of neg ative samples. The initial learning rate for the model is set at 0.004, and the training process incorporates an early stopping mechanism to prevent overfitting. Comparing with other methods, we strictly adhere to the hyperparameters and model configurations as specified in their original publications.

The performance of Struct2SL was assessed in comparison to the five aforementioned baseline models using three evaluation metrics: AUC, AUPR and F1-score. As illustrated in Table 3, Struct2SL outperforms all other baseline methods. Specifically, the AUC, AUPR, and F1-score for Struct2SL are 0.9812, 0.9798, and 0.9399, respectively. These metrics exceed those of the next best-performing model by 1.8 %, 0.9 %, and 3.4 %, respectively.

**Table 3:** Evaluation of the effectiveness among various techniques, with the best results emphasized in bold and the second-best results underlined.

Methods	AUC	AUPR	F1
DDGCN	0.8306±0.0052	0.2331±0.0078	0.8133±0.0065
TARSL	0.9258±0.0017	0.6134±0.0122	0.7078±0.0301
KG4SL	0.9427±0.0006	0.9504±0.0007	0.8883±0.0017
SLGNN	0.9635±0.0017	0.9710±0.0009	0.9089±0.0017
Struct2SL	**0.9812**±**0.0012**	**0.9798**±**0.0010**	**0.9399**±**0.0017**

The comparison results showed that the topological structure of the graph could propagate information and exploit the similarity of known synthetic lethal interactions, thereby improving the model performance. However, when predicting SL interactions, the baseline models primarily depend on the intrinsic attributes of genes to distinguish various relationships within the gene subgraph. Among these methods, TARSL meticulously analyzes the interaction relationships within the protein-protein interaction network. Yet, none of these methods consider the characteristic information of protein three-dimensional structures and protein sequences. The comparative analysis clearly demonstrates that Struct2SL integrates these factors, leading to its superior performance in generating gene embeddings for the prediction of SL interactions.

### 4.4 Ablation study

In the overall performance analysis, Struct2SL demonstrates a notable enhancement in forecasting the synthetic lethality within SL interactions. In this section, we perform experiments by omitting certain feature data during the model's data processing stage to assess its impact on the model's overall performance. To achieve this, we developed the relevant sub-models. The results of these ablation studies are presented in Table 4.

**Table 4:** Results of ablation experiments.

Methods	AUC	AUPR	F1
Struct2SL- 1024d	**0.9812**±**0.0012**	**0.9798**±**0.0010**	**0.9399**±**0.0017**
Struct2SL- 128d	0.9810±0.0006	0.9623±0.0008	0.9174±0.0008
${\mathrm{Struct}2\mathrm{SL}}_{\mathrm{no}\mathrm{ns}\mathrm{tru}\mathrm{ct}}\;$-128d	0.9755±0.0031	0.9496±0.0045	0.8956±0.0048
${\mathrm{Struct}2\mathrm{SL}}_{\mathrm{non}s\mathrm{eq}}\;$-128d	0.9804±0.0008	0.9596±0.0011	0.9081±0.0017
${\mathrm{Struct}2\mathrm{SL}}_{\mathrm{nonPPI}}\;$-128d	0.9691±0.0047	0.9489±0.0046	0.8940±0.0002

In prior protein sequence feature extraction, the resultant feature data had a dimensionality of 1024. To bolster the persuasiveness of the ablation study outcomes, this experiment reduced the protein sequence features to 128 dimensions, aligning them with the dimensions of the extracted protein structural features and PPI network data features, denoted as Struct2SL-128d. Building on this, each feature set was individually omitted, and the results were compared with the original model. It was evident that the omission of any feature set led to a decline in performance. This substantiates the contribution of the combination of protein structural, sequence, and interaction features to Struct2SL. Additionally, the performance of the model after dimensionality reduction also suffered, indicating that the original model's method of extracting protein sequence features possesses a stronger representational capacity compared to the reduced-dimension data, making a more significant contribution to the prediction of synthetic lethal gene pairs.

### 4.5 Case study

Recent studies have unveiled potential synthetic lethal interactions through scientific methods such as biological experiments, which can inform novel therapeutic strategies. For instance, the study titled " PLK1 inhibition induces synthetic lethality in Fanconi anemia pathway-deficient acute myeloid leukemia "[24], published on March 20, 2025, emphasized the significance of the PLK1-FANCD2 interaction in cancer metabolism. Similarly, the research "KLF5 loss sensitizes cells to ATR inhibition and is synthetic lethal with ARID1A deficiency"[25] published on January 8, 2025, highlighted the potential of targeting the KLF5-ARID1A axis in cancer cells. These findings have not yet been included into the experimental data of SynLethDB, meaning that these pairs of synthetic lethal genes were not present in previous experimental data. Therefore, they provide practical and persuasive cases for assessing whether the Struct2SL model can accurately predict potential synthetic lethal gene pairs.

Utilizing the Struct2SL framework, this study input the gene pairs PLK1-FANCD2 and KLF5-ARID1A into the model to evaluate its predictive capability for the synthetic lethality of these gene pairs. As previously mentioned, this framework integrates protein sequence, structure, and PPI network data to generate gene feature embeddings, which are then used for link prediction to identify potential synthetic lethal interactions. The prediction results are shown in Table 5.

**Table 5:** Analysis of two gene pairs (SL pairs).

gene pairs	prediction scores	predicted categories	actual categories
PLK1, FANCD2	0.99824	SL pairs	SL pairs
KLF5, ARID1A	0.84148	SL pairs	SL pairs

From the prediction results, the predicted scores for PLK1-FANCD2 and KLF5-ARID1A were 0.99824 and 0.84148, respectively, indicating that the Struct2SL model successfully predicted the synthetic lethality of the gene pairs. This is consistent with the results of the aforementioned studies, demonstrating the robustness and applicability of Struct2SL in the context of cancer research.

## 4 Conclusion

In this study, we present the Struct2SL model, which harnesses a multifaceted feature set to explore the latent potential of gene interactions at the protein level. By integrating data from protein sequences, Protein-Protein Interaction Networks, and the three-dimensional structures of proteins, the model augments the representation of gene interaction features, thereby facilitating more accurate predictions of potential synthetic lethal gene pairs. The Struct2SL model comprises four key stages: Initially, features are extracted from the 3D structures of proteins, their sequences, and interaction networks utilizing tools such as Alphafold2; subsequently, preliminary gene embeddings are generated by amalgamating data via the correlation between proteins and genes; thereafter, the SL graph is constructed to refine these gene embeddings; and finally, a multilayer perceptron is employed for predicting SL interactions.

Compared to current state-of-the-art methods for predicting SL interactions, Struct2SL exhibits superior performance in metrics such as AUC, AUPR, and F1-score. Furthermore, ablation studies have substantiated the substantial influence of integrating protein structural features, sequence data, and PPI features on the efficacy of the Struct2SL model. These findings suggest that the Struct2SL model represents a significant advancement in predicting the synthetic lethality of SL interactions, offering a novel and efficient computational approach for identifying SL gene pairs in cancer therapy, with the potential to catalyze the evolution of cancer treatment strategies.

In summary, the development and evaluation of the Struct2SL model provide a fresh perspective on predicting pairs of synthetic lethal genes based on protein features. Through the integration of multi-dimensional biological data, our model has realized notable enhancements in both predictive accuracy and interpretability. This approach not only optimizes the efficiency of feature extraction but also bolsters the model's ability to anticipate gene interactions, thereby furnishing robust support for the formulation of future cancer treatment strategies.

## CRediT authorship contribution statement

**Huang Yurui:** Writing – original draft, Validation, Software, Methodology, Formal analysis. **Yuan Ruzhe:** Writing – original draft, Validation, Software, Methodology, Investigation, Formal analysis, Data curation. **Li Yaxuan:** Software, Methodology, Investigation, Formal analysis. **Xing Zheming:** Software, Investigation, Data curation. **Li Junyi:** Writing – review & editing, Supervision, Resources, Project administration, Methodology, Funding acquisition, Formal analysis, Conceptualization.

## Authors' contributions

YH and RY designed the study, performed bioinformatics analysis and drafted the manuscript. All of the authors performed the analysis. JL conceived of the study, participated in its design and coordination and drafted the manuscript.

## Declaration of Competing Interest

The authors declare that they have no competing interests.

## Data Availability

All codes and data used in our experiments have been deposited at Availability: http://github.com/hyr-hit/Struct2SL.

## Footnotes

2 PyTorch Release 2.3.1 - Final RC is available, Release Announcements, PyTorch Developer Mailing List, https://dev-discuss.pytorch.org/t/pytorch-release-2–3-1-final-rc-is-available/2126.
